# An experimental and numerical study of the microstructural and biomechanical properties of human peripheral nerve endoneurium for the design of tissue scaffolds

**DOI:** 10.3389/fbioe.2022.1029416

**Published:** 2022-12-05

**Authors:** Liwei Yan, Ali Entezari, Zhongpu Zhang, Jingxiao Zhong, Jing Liang, Qing Li, Jian Qi

**Affiliations:** ^1^ Department of Microsurgery, Trauma and Hand Surgery, The First Affiliated Hospital of Sun Yat‐sen University, Guangzhou, China; ^2^ School of Biomedical Engineering, University of Technology Sydney, Ultimo, NSW, Australia; ^3^ School of Aerospace, Mechanical and Mechatronic Engineering, University of Sydney, Sydney, NSW, Australia; ^4^ School of Computing, Engineering and Mathematics, Western Sydney University, Penrith, NSW, Australia; ^5^ Guangdong Provincial Key Laboratory for Orthopedics and Traumatology, Guangzhou, China

**Keywords:** biomimetic scaffold design, nerve endoneurium, permeability, stiffness, nerve regeneration

## Abstract

Biomimetic design of scaffold architectures represents a promising strategy to enable the repair of tissue defects. Natural endoneurium extracellular matrix (eECM) exhibits a sophisticated microstructure and remarkable microenvironments conducive for guiding neurite regeneration. Therefore, the analysis of eECM is helpful to the design of bionic scaffold. Unfortunately, a fundamental lack of understanding of the microstructural characteristics and biomechanical properties of the human peripheral nerve eECM exists. In this study, we used microscopic computed tomography (micro-CT) to reconstruct a three-dimensional (3D) eECM model sourced from mixed nerves. The tensile strength and effective modulus of human fresh nerve fascicles were characterized experimentally. Permeability was calculated from a computational fluid dynamic (CFD) simulation of the 3D eECM model. Fluid flow of acellular nerve fascicles was tested experimentally to validate the permeability results obtained from CFD simulations. The key microstructural parameters, such as porosity is 35.5 ± 1.7%, tortuosity in endoneurium (*X* axis is 1.26 ± 0.028, *Y* axis is 1.26 ± 0.020 and *Z* axis is 1.17 ± 0.03, respectively), tortuosity in pore (*X* axis is 1.50 ± 0.09, *Y* axis is 1.44 ± 0.06 and *Z* axis is 1.13 ± 0.04, respectively), surface area-to-volume ratio (SAVR) is 0.165 ± 0.007 μm^−1^ and pore size is 11.8 ± 2.8 μm, respectively. These were characterized from the 3D eECM model and may exert different effects on the stiffness and permeability. The 3D microstructure of natural peripheral nerve eECM exhibits relatively lower permeability (3.10 m^2^ × 10^−12^ m^2^) than other soft tissues. These key microstructural and biomechanical parameters may play an important role in the design and fabrication of intraluminal guidance scaffolds to replace natural eECM. Our findings can aid the development of regenerative therapies and help improve scaffold design.

## Graphical Abstract

**Figure:**
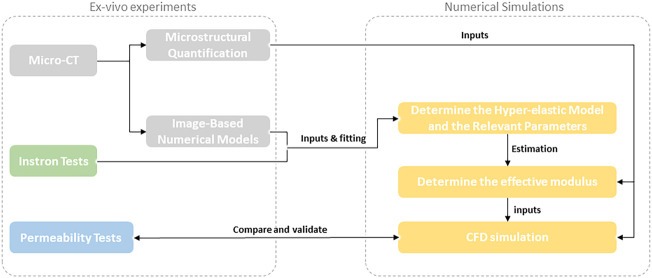
MicroCT was used to obtain high-resolution/high-contrast images of human peripheral nerve endoneurium extracellular matrix (eECM). A numerical model was constructed to investigate the microstructural and biomechanical properties of eECM.

## Introduction

Peripheral nerve injuries (PNIs) account for 3% of all traumatic patients and could cause partial or complete loss of function in damaged motor and sensory axons (Robinson, 2022). The most effective clinical strategy is graft transplantation, e.g., transplantation of autografts, which is currently used as the gold standard for nerve regeneration (Tiwari et al., 2022). The main advantage of autografts is their ability to largely mimic natural nerve characteristics, such as extracellular matrix (ECM) molecules, chemical and physical cues or topographic support and topological guidance (Wieringa et al., 2018). Nevertheless, the major limitations of autografts are their restrictive availability, donor site morbidity, and immunological rejection, which have prompted the development of alternative nerve repair strategies, such as nerve guidance conduits (NGCs) (Li et al., 2021).

Recent NGC studies have shown that conduits with proper micro- and nanostructures promote the regeneration of axons across a much longer gap of nerve defects (Li et al., 2021). This approach adopts macroscopic multichannel architectures (Pawelec et al., 2018) loaded with filaments (Yoshii et al., 2003) and unidirectional freeze-dried structures (Pot et al., 2015). Notably, NGC design aims to maximize the degree to which the conduit mimics the natural nerve architecture. According to previous studies, these biomimetic designs help promote nerve regeneration by enhancing the biomechanical behavior and porosity of the luminal wall using different materials (Zhu et al., 2018). However, the efficacy of NGCs is to a considerable extent limited to the defects with a size within a critical nerve defect, which is species-dependent and can range from 5 to 30 mm in animals (Yoshii et al., 2003). The key issue is usually attributed to inadequate formation of the ECM components and microstructures in the initial stages, which restricts Schwann cell (SC) migration and proliferation, thereby reducing the glial bands of the Büngner formation (Hoffman-Kim et al., 2010; Huang et al., 2022).

**FIGURE 1:**
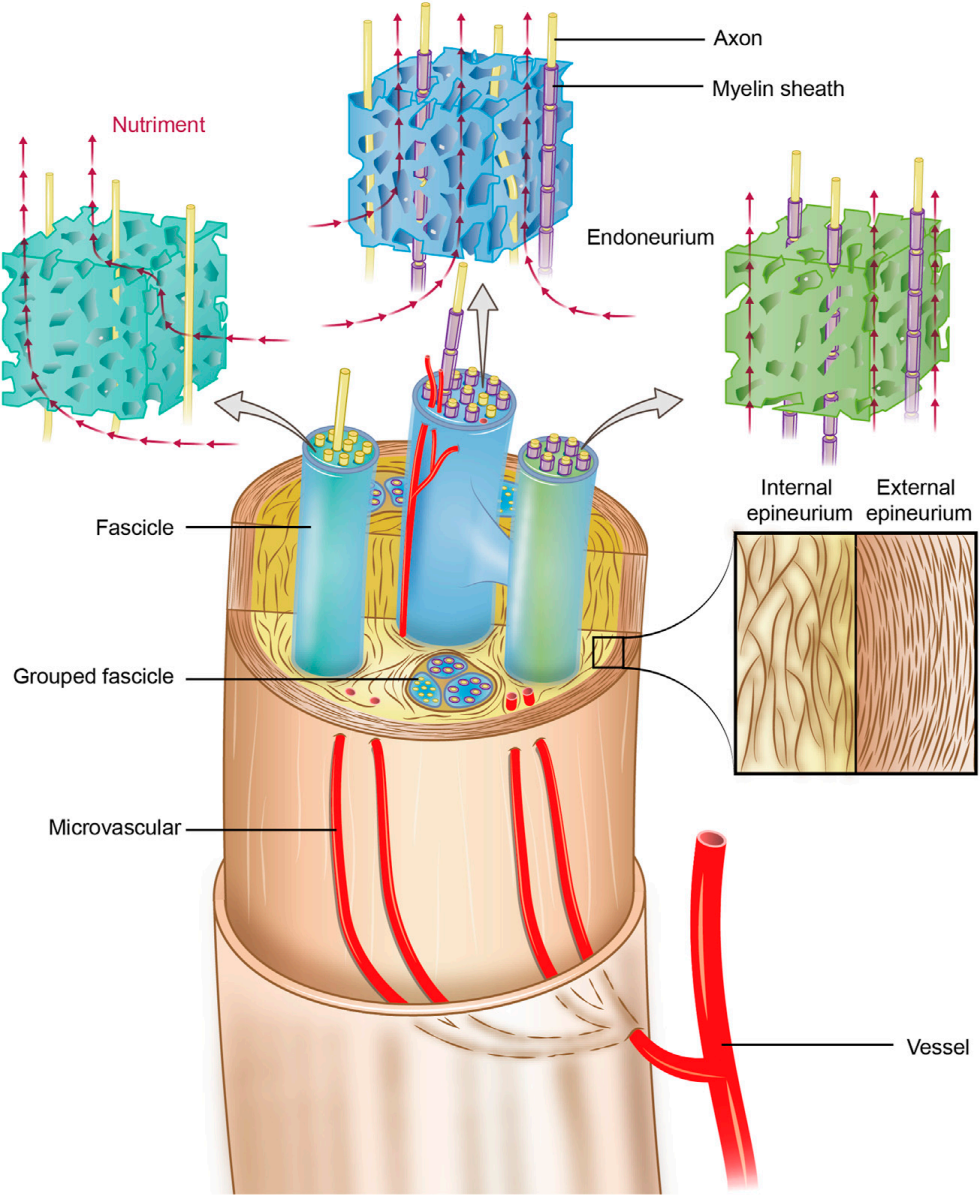
Schematic of the anatomy of the peripheral nerve ECM system. The endoneurium is an anisotropic porous structure that provides nutriment diffusivity longitudinally. The perineurium surrounds groups of axons to form fascicles, and the epineurium binds individual nerve fascicles into a nerve trunk. The internal microvasculature runs longitudinally within the internal epineurium and connects with the external vasculature at various points; thus, it plays a particularly vital role in nerve healing and repair after injury/damage.

To overcome these shortcomings, researchers have sought to design a scaffold with high similarity to the natural microarchitectures of peripheral nerves, especially the microstructure of the endoneurium (Chen et al., 2019; Yang et al., 2021). To realize this task, the first step is to characterize the microstructures and biomechanical properties of the peripheral nerve endoneurium ECM (eECM). The peripheral nerve ECM system is categorized into an external epineurium, an internal epineurium, fascicles and an endoneurium (Schmidt and Leach, 2003; Tiwari et al., 2022). The anatomy of the peripheral nerve ECM is illustrated in Figure 1, which shows that the perineurium surrounds groups of axons to form fascicles, and the epineurium binds individual nerve fascicles into a nerve trunk. The internal epineurium contains a complex microvascular network that plays a vital role in nerve healing and repair after injury/damage.

The biomimetic design of the scaffold architecture is a critical yet complex process requiring the appropriate selection and control of multiple parameters, such as mechanical properties, biodegradation, biocompatibility, pore architecture, porosity, surface properties, and, more importantly, permeability (Singh et al., 2018a; Gotti et al., 2020). Notably, the permeability of porous scaffolds substantially affects cell recruitment, differentiation, and proliferation and tissue regeneration after implantation, which determines the transport of oxygen/nutrients and removal of metabolites (Santos et al., 2020). For example, at a given cell seeding density, high permeability scaffolds will enhance bone regeneration (Mitsak et al., 2011). However, an overly high permeability might be a disadvantage due to an excessive flow rate, which could cause cell washout, thus resulting in a lower tissue growth rate. On the other hand, a permeability lower than the desired value would lead to low nutrient supply or even hypoxia to cells, thus hindering tissue growth. Hence, there is always a range of desired permeabilities as found in different tissues throughout the body (Gomez et al., 2016; Chao et al., 2021). Nevertheless, an optimal permeability of peripheral nerve scaffolds for facilitating nerve regeneration has remained largely unclear.

In a previous study, we proposed to implement an iodine and freeze-drying enhanced microscopic computed tomography (micro-CT) imaging technique that would enable us to obtain the high-resolution microstructure of the human peripheral nerve, which allowed us to construct a baseline model for numerical characterization *in silico* here (Yan et al., 2017a; Yan et al., 2019; Almeida and Bartolo, 2021). The present study aims to investigate the key microstructural characteristics and permeability of the human peripheral nerve eECM, in which the effects of porosity, tortuosity, surface area-to-volume ratio (SAVR) and pore size on the stiffness and permeability are explored using experimental and numerical approaches. The contributions of this study are as follows (Robinson, 2022): we first applied micro-CT to obtain high-resolution/high-contrast images of human peripheral nerve endoneurium extracellular matrix (eECM) (Tiwari et al., 2022); A numerical model was constructed to investigate the permeability of eECM (Wieringa et al., 2018); We anticipate to provide a fundamental understanding and insights into the design of scaffolds for regenerating peripheral nerves.

## Materials and methods

### Harvesting of human peripheral nerve specimens

All human experiments were performed using the well-established protocol approved by the institutional review boards of the first Affiliated Hospital of Sun Yat‐sen University, China in accordance with the Declaration of Helsinki. In this study, we adopted characteristic human nerves excised from the upper or lower limbs of amputation patients at the first Affiliated Hospital of Sun Yat‐sen University. All donors provided specific consent for the use of their donated tissues for teaching and scientific research purposes. We selected three different proximal median nerve as the microCT study. The remaining nerve specimens were used for tensile test and others experiments.

### Specimen preparation and protocol for Micro-CT scanning

Micro-CT was used to acquire high-resolution images from the preprocessed samples using the iodine and freeze-dry (IFD) method described previously (Yan et al., 2017a; Yan et al., 2019). Preprocessing steps are briefly summarized here for completeness (Robinson, 2022). Fresh nerves were immersed in 50% Lugol’s solution (Sigma-Aldrich, St. Louis, MO, United States, 62,650–100 ML-F; 0.5%–1% potassium iodide and 0.25%–0.5% iodine) for 3 days in a 50-ml centrifuge tube and manually shocked as appropriate. (Tiwari et al., 2022). The samples were placed into liquid nitrogen for 2 min and stored in a drying machine (LABCONCO, FreeZone 6 Plus, United States) for 7 days.

The micro-CT scanning protocol (μ50, SCANCO Medical AG, Bassersdorf, Zurich, Switzerland) was established as follows: holder, 9 mm; energy/intensity, 55 kVp, 72 μA, 4 W; filter, 0.1 mm Al; BH, Plexi (PMMA); FOV/diameter, 10.2 mm; integration time, 1,500 m s; average data, 3; voxel size, 3 μm; and sample pixel, 3,400 × 3,400. Finally, we obtained at least 250 sectional CT images for each sample. After micro-CT scanning, we used the same samples to acquire scanning electron microscopy (SEM) images. The samples were cut transversely into 2-mm-thick sections with a razor blade. After platinum coating, they were examined by SEM (FEI QUANTA 200, Netherlands).

### Masson’s trichrome staining for histological analysis

The human nerve samples (*n* = 3) were immersed in an optimum cutting temperature compound (O.C.T. compound; Sakura of America, Hayward, California, United States). Transverse sections were cut using a cryostat (CM3050s; Leica, Wetzlar, Germany) to a thickness of 50 mm and mounted on microscope slides. They were then subjected to Masson trichrome staining and observed under a light microscope. We collected four photographs from randomly selected fields in each specimen and then measured the cross-sectional area of the pore using Image-Pro Plus software (Media Cybernetics, Bethesda, Maryland, United States).

### Micro-CT image segmentation

Voxel-based models were processed using a commercial software package (ScanIP 2017; Simpleware, Exeter, United Kingdom). A sequence of 250 slices of non-smoothed transverse-plane micro-CT images was imported into the program in a computer [Intel(R) Core (TM) i7-4930K CPU @ 3.40 GB memory]. The threshold for binarizing the images (i.e., segmentation into pores and endoneurium tissue) was set to the minimum value between the two peaks in the grayscale histogram. Two steps are required prior to segmentation: the first is image refinement, in which we used the ScanIP smoothing module plus the mean filter algorithm to remove noise and gradients; and the second is cropping to preserve only the region of the data containing the endoneurium microstructure. Thus, cubic shapes of 200×200×200 voxels for each sample were generated. As a result, two hundred 2D images were obtained for each region of interest (ROI). In the current study, at least three ROIs at different locations of each individual sample were modeled for characterization.

### Reconstruction of the 3D microstructure

The acquired grayscale images were segmented into binary images using a grayscale threshold, where the pixels with grayscale values greater than the preselected threshold (denoted as TV) were labeled as the endoneurium (solid phase), and the pixels with grayscale values less than the TV were classified as background (pore phase). Once this process was performed for each 2D image, the endoneurium region and pore region were interconnected across the third dimension to reconstruct the 3D models.

### Selection of the representative volume element

The RVE is commonly used to characterize the effective properties of any porous material, which should be selected appropriately to determine the effective behavior and ensure efficient computational modeling.

### Quantification of microstructural parameters

The key microstructural parameters of the endoneurium, such as the porosity, tortuosity, SAVR and pore size, were quantified using the ScanIP 2017 measurement module. Porosity was calculated as the fraction of pore phase volume to the total volume. Tortuosity represents a geometric parameter describing the extent of twisted interconnected paths through the volume. In this study, the tortuosity of both the pore and endoneurium phases was measured using a centerline module based upon a computed path formed by centroids of each interconnected region as the same phase on each image. We used the shortest route module in ScanIP to measure the tortuosity in different directions. The SAVR measures the relative of the endoneurium phase surface area to the total volume. The pore size was obtained by applying an object separation technique in the microstructures with a watershed algorithm. After the pore/endoneurium phase was separated into a group of individual particles/pores, the volume of each particle/pore was converted to an equivalent spherical diameter (ESD) for plotting particle/pore size distribution histograms (Gentile et al., 2012).

### *Ex vivo* experimental setup

Human fascicle samples (n=6) were carefully isolated within 24 h after harvest from the donors and immersed in a 9% normal saline solution to prevent dehydration during preparation. The diameters of the isolated fascicle samples were measured before the test to calculate the cross-sectional area, in which the average diameter was 2.02±0.78 mm. One end of the sample was wrapped in sandpaper to prevent slippage and mounted on an Instron machine (Instron 3343, INSTRON COMPANY, United States) for the uniaxial tensile test. To precondition the specimens, each specimen was suspended by the top clamp and allowed to hang freely for 2 min. The fascicles were considered under no external loads and fully relaxed, and the length of the fascicles was measured and used as the gauge length. The tensile test was then performed with a 10 mm/min strain rate until complete rupture.

### Microstructural finite element modeling

Microstructural finite element (μ-FE) models of endoneurium RVEs (n=3) were constructed and exported using ScanIP N-2018.03 (Simpleware, Exeter, United Kingdom) based on the micro-CT data. The endoneurium was segmented with a proper threshold to ensure that the exported models had the same porosity as the measurements from histological images. The μ-FE models were meshed in linear tetrahedral elements with an average degree of freedom of 1,642,833 and then imported into ABAQUS 6.13 (SIMULIA, Providence, RI, United States), where each μ-FE model of RVEs was placed between the two parallel rigid plates to replicate the tensile tests. One of the rigid plates was fixed to the base, and the other plate was subjected to a load that would generate stress equivalent to that induced by the experimental load on the RVE surface.

Experimental loads beyond the toe region were randomly chosen and converted to corresponding loads on the RVEs as follows:

$$F_{RVE}=\frac{F_{Macro}A_{RVE}}{A_{Macro}}$$ (1)

where $F_{Macro}$ and $F_{RVE}$ are the load on the endoneurium (macrolevel) and the corresponding load on the RVE (sublevel), respectively; and $A_{Macro}$ and $A_{RVE}$ are the areas of the endoneurium (macrolevel) and RVE (sublevel), respectively.

### Characterization of the mechanical properties of fascicles

A μ-FE analysis was performed to inversely identify the optimal set of material parameters with the best possible match to the characteristics of the human fascicles. The four widely used hyperelastic constitutive models for describing biological soft tissues (Ogden, Yeoh, Marlow, and Mooney-Rivlin) were used here to characterize the hyperelastic properties. To assess the material properties of the μ-FE models that provide the best fit to the experimental data obtained from the uniaxial tensile tests, the minimum mean square error based on the regression curves was calculated. The third-order Yeoh’s model best fit the test data and displayed the highest stability. Thus, the third-order Yeoh’s hyperelastic model was adopted and carefully adjusted until the resulting effective modulus of the bulk RVE in the z direction (i.e., the longitudinal direction of the nerve) matched the linear phase of the obtained J-shaped stress-strain curve.

Then, the identified material properties were further adopted to determine an effective modulus in the x and y directions (transverse directions of the nerve). The effective modulus was derived using the following equations (Entezari et al., 2019):

$$E_{x}=\frac{F_{x}\times l_{x}}{u_{x}\times A_{yz}}$$ (2)

$$E_{y}=\frac{F_{y}\times l_{y}}{u_{y}\times A_{xz}}$$ (3)

$$E_{z}=\frac{F_{z}\times l_{z}}{u_{z}\times A_{xy}}$$ (4)

where E , F and l denote the effective young’s modulus, the applied force and the initial length of the sample in the direction of the corresponding force, respectively; A represents the area of the loaded cross-section; u denotes the displacement; and subscripts x , y , and z indicate the direction of the parameter.

### Computational fluid dynamics analyses

The micro-CT-based models of endoneurium RVEs ( n=3 ) with dimensions of 300×300×300 µm were created in ScanIP. Notably, the most critical step in creating the micro-CT-based CFD models of fascicle tissue is to estimate a reasonable threshold range for segmentation of the soft tissue. For this purpose, arbitrarily segmented 2D images from each sample were compared with the random images of histology to ensure that the surface area of the soft tissue was the same in both the segmented and histological images.

In the CFD simulation, the fluid was assumed to fully fill the void space in the endoneurium RVEs; therefore, the inverse model of each RVE was first generated in ScanIP to represent the fluidic phase. Then, each RVE model was imported into the Fluid Flow (Fluent) module in ANSYS Workbench 2019 for the permeability analyses. A constant flow rate ( Q ) and zero static pressure were applied for the inlet and the outlet, respectively. Steady-state Navier–Stokes equations were implemented, and no-slip conditions were considered for the walls of the RVE models. The fluidic phase was considered to be water that was incompressible and homogeneous with a density and dynamic viscosity of 1,000 kg m^−3^ and 1 Pa s × 10^–3^ Pa s, respectively. The resulting decrease in pressure ( ∆P ) through the fascicle RVEs was used to calculate the effective permeability from Darcy’s law, which is defined by the following equation:

$$k=\frac{\mu L}{A}\frac{Q}{∆P}$$ (5)

where k denotes the effective permeability (m^2^), L is the thickness (m) of RVE, A is the area of the RVE cross-section (m^2^), μ is the dynamic fluid viscosity (Pa. s), and ∆P is the pressure gradient (Pa) in the fascicle RVE when fluid flows at a rate of Q (m^3^ s^−1^) through the endoneurium model. Since Darcy’s law is valid for Reynolds numbers less than one, a small flow rate ( Q ) was applied as the inlet boundary condition.

### Permeability tests

The decellularized human nerve samples were bought from Guangzhou Zhongda Medical Devices Company (Guangzhou, Guangdong Province, China). The nerve fascicles were separated under the microscope due to the experiment in this part. The permeability of the fascicle samples was measured using the falling head method (Zhang et al., 2019a). Using Darcy’s law, the permeability of a sample can be derived. The initial heights L1 at t0 and as the final height L2 at t1 were recorded in the tests. The values of L1 and L2 remained the same in each test to simplify the calculation. The hydraulic conductivity K of the sample was calculated from the equation of Darcy’s law:

$$K=\frac{aH}{A\left(t1-t0\right)}\mathrm{Ln}\left(\frac{L1}{L2}\right)$$ (6)

where a is the cross-sectional area of the standpipe, A is the cross-sectional area of the sample, and H is the height of the sample.

The permeability k of the tested sample was calculated using the following equation:

$$k=\frac{K\lambda }{\rho g}$$ (7)

where λ is the dynamic viscosity coefficient of water, ρ is the density of water, and g is the acceleration of water due to gravity. The parameters used in the permeability tests are summarized in Table 1.

**TABLE 1:** Parameters and their values used in the permeability tests.

Parameters	Sample 1	Sample 2	Sample 3	
Average height of the sample (H)	1.00 m × 10^−1^ m	1.00 m × 10^−1^ m	1.00 m × 10^−1^ m	
Cross-section of the standpipe (a)	2.16 m^2^ × 10^−4^ m^2^	2.16 m^2^ × 10^−4^ m^2^	2.16 m^2^ × 10^−4^ m^2^	
Cross-section of the sample (A)	2.74 m^2^ × 10^−6^ m^2^	2.52 m^2^ × 10^−6^ m^2^	2.37 m^2^ × 10^−6^ m^2^	
Dynamic viscosity coefficient of water (u)	1.00 Pa^.^s × 10^−3^ Pa^.^s	1.00 Pa^.^s × 10^−3^ Pa^.^s	1.00 Pa^.^s × 10^−3^ Pa^.^s	
Density of water (*p*)	1.00 kg/m^3^ × 10^3^ kg/m^3^	1.00 kg/m^3^ × 10^3^ kg/m^3^	1.00 kg/m^3^ × 10^3^ kg/m^3^	
Gravity acceleration (g)	9.8 m/s	9.8 m/s	9.8 m/s	
L1	1.46 m	1.46 m	1.46 m	
L2	1.44 m	1.44 m	1.44 m	
t	350 s	1,196	532	
K	3.11 × 10^−5^	9.89 × 10^−6^	2.36 × 10^−5^	
*k* [m^2^]	3.17 × 10^−12^	1.01 × 10^−12^	2.41 × 10^−12^	

K , hydraulic conductivity; k , permeability.

### Statistical analysis

All the experiments were performed in triplicate, with a minimum sample size of n=3 . The results of the quantitative experiments were expressed as the mean ± standard deviation. All analyses were carried out using GraphPad Prism 8 with 2-tailed Student’s t-tests and one-way ANOVAs. A P value less than 0.05 was considered statistically significant.

## Results

### Micro-CT imaging and 3D reconstruction of the human peripheral nerve endoneurium

**FIGURE 2:**
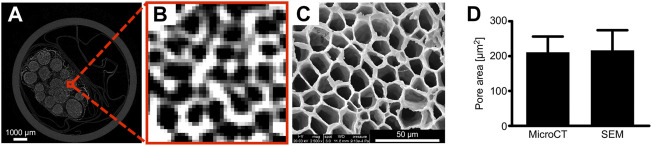
Human peripheral nerve Micro-CT images. Compared with SEM, micro-CT images can clearly distinguish the endoneurium microstructure. The measurement area of the pore compared with SEM showed that micro-CT 2D images are authentic. **(A)** Micro-CT images of the nerves. **(B)** Micro-CT images of the endoneurium. **(C)** SEM images. **(D)** Comparison of the pore areas. *n* = 10; the scale bars represent 1,000 μm and 50 μm.

We employed an iodine and freeze-drying method to preprocess human peripheral nerve samples and obtain high-resolution micro-CT images (Figure 2A); and the SEM images were also acquired for comparison with the same sample (Figure 2C). We measured the area of the endoneurium by comparing the micro-CT scan with the SEM image to confirm that the micro-CT scan generates a sufficiently accurate image (Figure 2D). After the micro-CT scan, some pixels were not captured in the images properly, thereby highlighting the importance of the image threshold for the subsequent segmentation step.

One of the most critical steps in creating micro-CT-based models of nerve tissue for microscopic finite element analysis (μ-FEA) and microscopic computational fluid dynamics (μ-CFD) modeling is to estimate a reasonable threshold range for segmentation of the eECM. For this purpose, arbitrarily segmented 2D images from each sample were compared with some random images of histology to ensure that the surface area of the eECM was the same in both segmented and histological images (Chen et al., 2015a). Here, we adopted the surface area rate obtained from the peripheral nerve 2D histological images to correct the segmentation of micro-CT images (Chen et al., 2015b).

**FIGURE 3:**
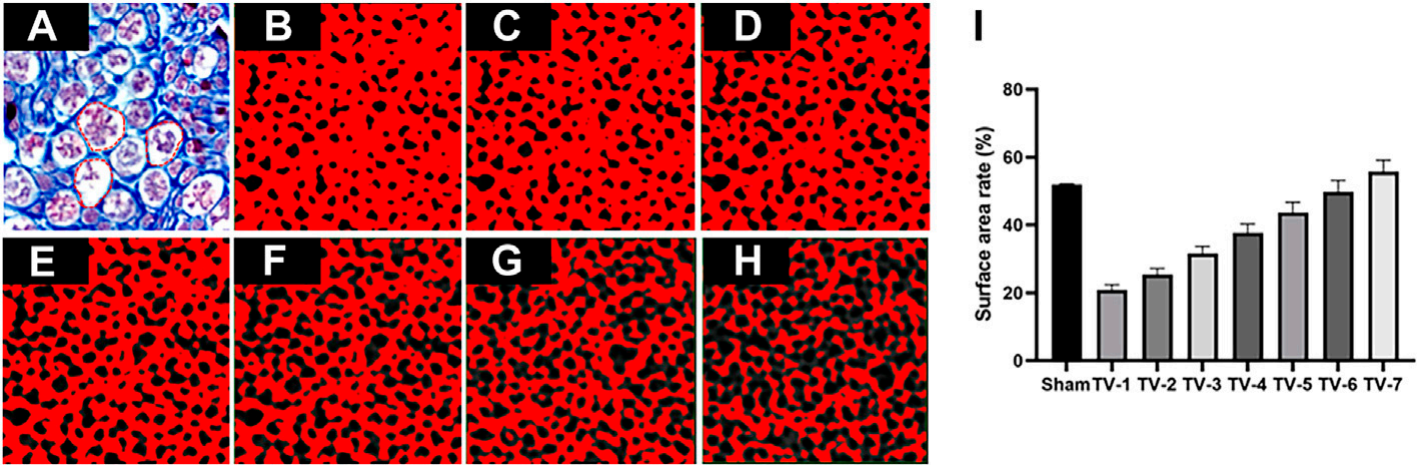
Representative TVs used to segment eECM micro-CT images. **(A)** Masson’s trichrome staining and binary image obtained by applying a TV of **(B)** 25, **(C)** 26, **(D)** 27, **(E)** 28, **(F)** 29, **(G)** 30 and **(H)** 31. **(I)** Surface rate results. The red dotted line represents the pore of endoneurium. *n* = 3.

We obtained a 2D histological image (Figure 3A) with a surface area rate of 51.86±0.25% (Figure 3I). The binarized endoneurium images are shown in Figures 3B–H. Using the threshold value (TV) to segment endoneurium micro-CT images, 200×200 -pixel eECM micro-CT images were used to obtain a preliminarily defined range of TVs. A number of grayscale values within a limited range close to 30 were selected for segmentation, specifically, TV = 25, 26, 27, 28, 29, and 31, to evaluate the sensitivity of the surface area rate with respect to variation in the TV. The 2D micro-CT images of the surface area rate after applying the aforementioned TVs are illustrated in Figure 3I, which shows a grayscale image from the stack and the resulting binary images generated when different TVs were applied for segmentation. The results indicate that the surface area rate was closest to that of the histological image when the TV was 30. To reduce the computational load, we used 3D porosity to select the RVE of eECM samples.

### Key parameters of the 3D endoneurium extracellular matrix microstructure

The key 3D microstructural parameters, such as pore size, eECM thickness, porosity, tortuosity and SAVR, were calculated ( n=3 ) and are summarized in Table 2. For microstructural complexity, the tortuosity of the eECM and the pore phase may not be the same. Tortuosity cannot be less than 1, and microstructures with more twisted and complex features result in a higher tortuosity value. In the current study, the tortuosities of both the endoneurium tissue and the pore space were quantified, and the results are presented in Table 2; t both the tortuosities for endoneurium and pores are smaller in the z direction than those in the other directions. The tri-directional tortuosity of each group indicates that the endoneurium microstructure has an anisotropic porous structure. Therefore, tortuosity can also be determined based on the transport equation that governs the permeability in the entire media of the structural phase (Chen et al., 2015a).

**TABLE 2:** 3D microstructural parameters of endoneurium ECM.

3D microstructural parameters	Values
Pore size	11.8 ± 2.8 μm
Endoneurium thickness	11.4 ± 3.1 μm
Porosity	35.5 ± 1.7%
Tortuosity	
Endoneurium-X	1.26 ± 0.028
Endoneurium-Y	1.26 ± 0.020
Endoneurium-Z	1.17 ± 0.03
Pore-X	1.50 ± 0.09
Pore-Y	1.44 ± 0.06
Pore-Z	1.13 ± 0.04
SAVR	0.165 ± 0.007 μm^−1^

3D, three-dimensional; ECM, extracellular matrix; SAVR, surface area-to-volume ratio.

### Tensile test

In the present experimental study, we investigated the stress-strain characteristics of the human peripheral nerve fascicles ( n=6 ). The tensile tests showed that the peripheral nerve was easily extensible in the initial stage followed by stiffening, which exhibited a typical J-shaped force-displacement curve similar to those of other soft tissues (El-Bakary et al., 2019). The ultimate loads were recorded from 3.39 to 35.93 N, depending on the diameter of the samples, as shown in Table 3.

**TABLE 3:** Sample sizes and ultimate loads.

Sample ID	Sample 1	Sample 2	Sample 3	Sample 4	Sample 5	Sample 6
Diameter [mm]	3.08	1.72	2.35	3.10	1.22	1.00
Ultimate Load (N)	28.97	7.44	29.59	35.93	6.85	3.39

**FIGURE 4:**
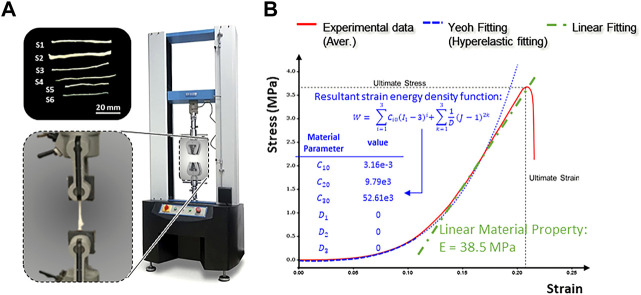
Tensile testing of human peripheral nerve fascicles. **(A)** Experimental setup. **(B)** Typical stress-strain curve obtained after tensile testing of the peripheral nerve fascicles.

Figure 4 shows the stress-strain curve based on the average data for the 6 samples tested, which all exhibited characteristic J-shaped nonlinear behavior. The curve has a large initial toe region, reaching a strain of approximately 13% under minimal stress. Much of the strain within the toe region may be due to relaxation of the slack fibrils within the fascicles. Notably, the nerve strain induced by physiological motion varies with the anatomical position of the nerves and the angle of joint flexion but is generally within the toe region of the J curve (Kwan et al., 1992). A linear trend was identified in the stress-strain curve after the toe region, where the slope steadily increased in the initial stage and then remained constant with a young’s modulus of 38.5 MPa until the sample ruptured.

A linear regression analysis of the numerical simulation and experimental test results showed an $R^{2}$ value greater than 0.83. However, other scientific studies have reported that the elastic modulus of intact human nerves is 8−16 MPa (Chiono and Tonda-Turo, 2015). Our results differed from those of the previous studies mainly because the area of the measured sample was different.

### FE simulation of Young’s modulus

**FIGURE 5:**
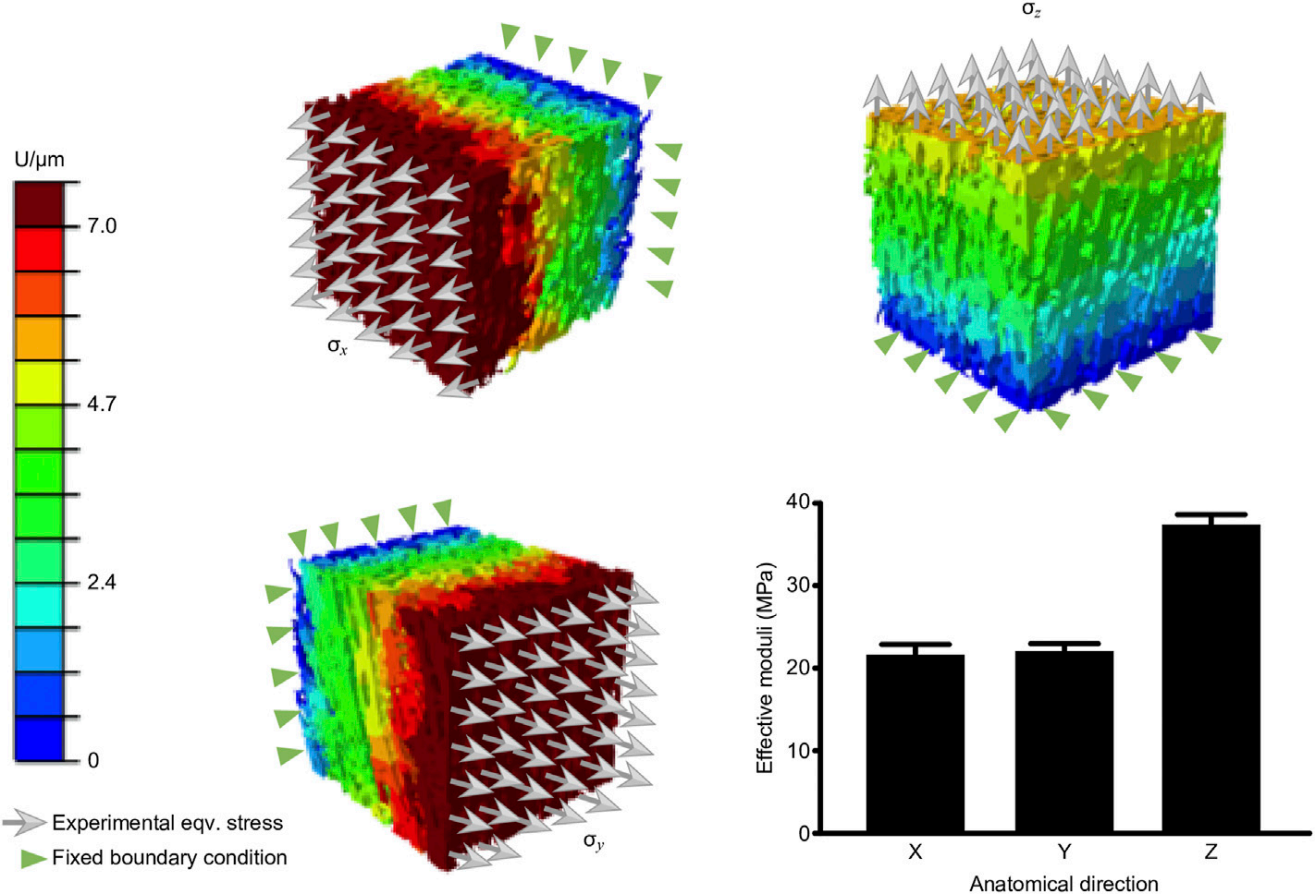
Computed elastic moduli in the three principal directions.

The μ-FE simulation was performed in the linear phase of the J curve because this region is where pathological stress/strain and thresholds of damage and/or rupture of the nerve are commonly considered. The resulting longitudinal ( z direction) effective young’s modulus ( E ) was 37.46±1.20 MPa, which was very close to the experimental effective value of E in the linear phase ( 38.5 MPa). The simulated effective E of the lateral directions ( x and y directions) were 21.69±1.22 MPa and 22.19±0.71 MPa, respectively. According to the present results, human nerve fascicles are transversely isotropic materials; and their principal direction is along the longitudinal direction, which has higher mechanical properties than those in the transverse directions. Figure 5 summarizes the simulation results.

### Computational fluid dynamic simulation of permeability

**FIGURE 6:**
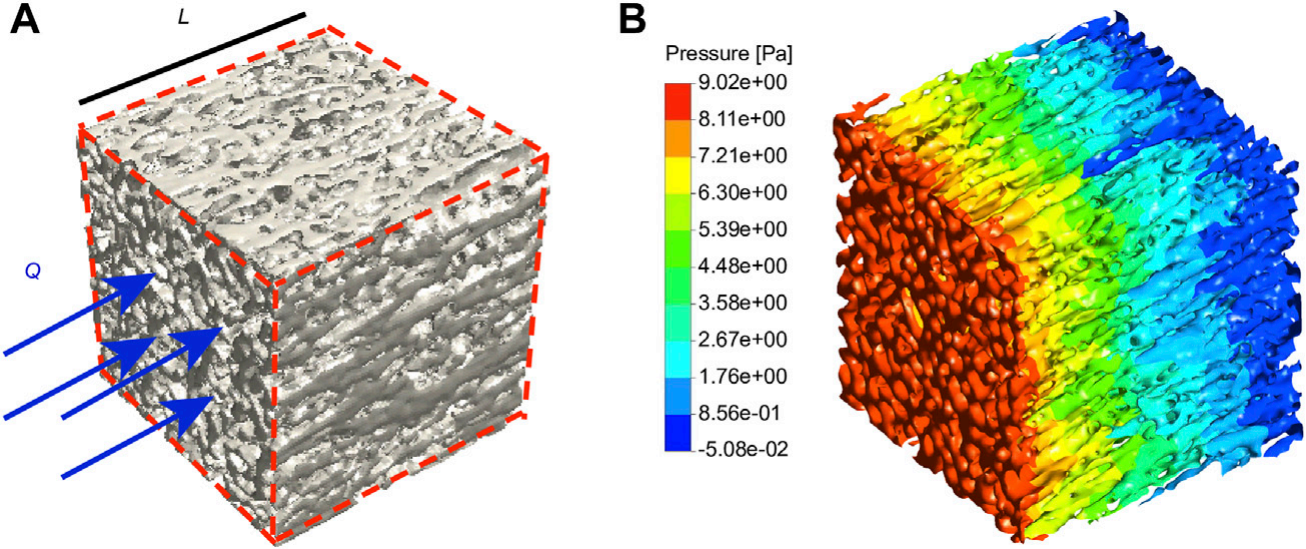
Fluid flow simulation. **(A)** The eECM RVE considered in the CFD simulations is subject to a mass flow rate of *Q*. **(B)** The contour of the fluid pressure in the eECM RVE is obtained from CFD simulations. *n* = 3.

Figures 6A-B shows the contour of fluidic pressure in an eECM RVE subject to the mass flow rate of $8.75\times 10^{-9}$ (kg/s). The decreases in the pressure of each RVE ( n=3 ) were used to calculate the permeability of the eECM according to Darcy’s law. Table 4 summarizes the permeability of these three reconstructed eECM specimens (RVE) calculated in the μ-CFD analyses, with a value of $3.10\pm 1.91\times 10^{-12}$ m^2^.

**TABLE 4:** The permeability of the endoneurium ECM RVEs computed using CFD simulations.

Permeability, *k* [m^2^]				
Sample 1	Sample 2	Sample 3	Average	SD
3.29 × 10^−12^	2.84 × 10^−12^	3.18 × 10^−12^	3.10 × 10^−12^	1.91 × 10^−13^

ECM, extracellular matrix; RVEs, Representative Volume Elements; CFD, computational fluid dynamic; k , permeability.

### Permeability tests *ex vitro*

Permeability, a quantitative measure of cell and nutrient migration through pore channels, has been considered a key criterion for the design of porous scaffolds. Three decellularized human nerve samples were used for the experimental tests, and the results are summarized in Table 5. The average endoneurium permeability value was $2.19\pm 1.09\times 10^{-12}$ m^2^.

**TABLE 5:** Parameters and their values used in the permeability tests of the three groups.

Parameter	Sample 1	Sample 2	Sample 3
Average height of the sample (H)	1.00 m × 10^−2^ m	8.00 m × 10^−3^ m	1.00 m × 10^−2^ m
Cross-section of the standpipe (a)	3.58 m^2^ × 10^−3^ m^2^	3.58 m^2^ × 10^−3^ m^2^	3.58 m^2^ × 10^−3^ m^2^
Cross-section of the sample (A)	1.42 m^2^ × 10^−5^ m^2^	1.24 m^2^ × 10^−5^ m^2^	5.20 m^2^ × 10^−5^ m^2^
Dynamic viscosity coefficient of water (u)	1.00 Pa^.^s × 10^−3^ Pa^.^s	1.00 Pa^.^s × 10^−3^ Pa^.^s	1.00 Pa^.^s × 10^−3^ Pa^.^s
Density of water (*p*)	1.00 kg/m^3^ × 10^3^ kg/m^3^	1.00 kg/m^3^ × 10^3^ kg/m^3^	1.00 kg/m^3^ × 10^3^ kg/m^3^
Gravity acceleration (g)	9.8 m/s	9.8 m/s	9.8 m/s
L1	0.45 m	0.45 m	0.45 m
L2	0.425 m	0.425 m	0.425 m
t			
#1	950 S	1440 S	5400 S
#2	890 S	1540 S	4460 S
#3	520 S	2130 S	5480 S
K			
#1	1.51 × 10^−4^	9.17 × 10^−5^	7.29 × 10^−6^
#2	1.61 × 10^−4^	8.57 × 10^−5^	8.23 × 10^−6^
#3	2.77 × 10^−4^	6.20 × 10^−5^	7.18 × 10^−6^
*k* [m^2^]			
#1	1.55 m^2^ × 10-^11^ m^2^	9.35 m^2^ × 10^−12^ m^2^	7.44 m^2^ × 10^−13^ m^2^
#2	1.65 m^2^ × 10^−11^ m^2^	8.75 m^2^ × 10^−12^ m^2^	9.09 m^2^ × 10^−13^ m^2^
#3	2.83 m^2^ × 10^−11^ m^2^	6.32 m^2^ × 10^−12^ m^2^	7.33 m^2^ × 10^−12^ m^2^

K , hydraulic conductivity; k , permeability.

## Discussion

When provided with a suitable ECM microenvironment, the peripheral nerve has an intrinsic capacity for functional recovery over time (Du et al., 2018; Philips et al., 2018). ECM functions both as a material for supporting the microstructure and as a regulator of cell differentiation, signal transduction, proliferation, morphogenesis, and remodeling in the host (Gilbert et al., 2006). Autografts provide a suitable ECM microenvironment, which currently remain the clinical gold standard for repairing nerve defects (He et al., 2015). Their success has been attributed to the presence of an intact ECM microarchitecture and viable SCs, which concomitantly provide ideal support for orientated axon regeneration (Singh et al., 2018b). However, nerve autografting has inherent flaws: specifically, a secondary surgery at the donor site is required, dysfunction of distal organ may occur in the donor nerve, and the length and/or diameter of the autologous nerve may be insufficient for effective reconstruction (Kehoe et al., 2012). Another major shortcoming of autografts is the microstructural mismatch of nerves from different anatomic sites, which may lead to inappropriate and incomplete reinnervation and subsequently poor functional recovery (Bain et al., 1989). In previous animal experiments, we varied the fascicle spatial position to explore the effect of precise fascicle matching on the effectiveness of nerve function recovery and found that a microstructural mismatch causes poor functional recovery (Yan et al., 2017b).

Compared with autografts, decellularized allografts well confirm the role of ECM microarchitecture in nerve regeneration (Zaminy et al., 2021). In this approach, cells are removed from the decellularized allograft but the 3D microstructure and composition of the ECM are preserved (Sondell et al., 1998). The preserved ECM components create a milieu similar to peripheral nerve tissue for schwann cell adhesion and proliferation (Su et al., 2022). Some studies show that removing the factors inhibiting axonal growth from ECM will enhance the effect of acellular nerve allograft (ANA) (Li et al., 2022). Our team developed a ANA product approved by the Chinese Food and Drug Administration (CFDA) (Guangzhou Zhongda Medical Devices Company, Guangzhou, Guangdong Province, China; Approval No. (2012) 3460641), and it has been used in clinical repair over the years. The treatments show that ANA facilitate safe and effective reconstruction of distal nerve defects with a length of 10–60 mm in human patients (Zhu et al., 2017). In the literature, another study used allografts to repair nerve defects with gaps of 30–70 mm in the clinic (Safa and Buncke, 2016). Despite these clinical successes, the reason for the limited effectiveness of ANAs in repairing proximal mixed nerve defects remains largely unknown (Thomson et al., 2022).

ANA’s materials are mainly from cadavers, which limits its widespread application (Jiang et al., 2022). When ANA is used to repair long or large nerve defects, neural cell necrosis may occur due to insufficient early vascularization, especially when the graft is too thick and revascularization does not reach the center of the graft (Ray and Mackinnon, 2010). Consistent with the results of clinical and fundamental experimental studies, we speculate that ANAs do not form an optimal microenvironment for regeneration (Thomson et al., 2022). The bionic scaffold not only requires sufficient ECM components, but also provide topological guidance by mimicry of microstructure of natural eECM (Rao et al., 2021; Tiwari et al., 2022). The biomechanical properties, such as stiffness and permeability, can be modulated during the design and fabrication of NGCs to replicate the physical cues and structural characteristics of natural ECM (Wegst et al., 2015; Yang et al., 2021).

Importantly, natural biomaterials are commonly arranged in rather sophisticated hierarchical architectures, and their components exhibit extraordinary biomechanical characteristics. In the literature, three factors were found to be critical for the development of new scaffolds: chemical composition, nano/microstructure and biomechanical properties (Sun et al., 2020). As exemplified by the development of peripheral nerve repair grafts, the first step is to analyze the microstructural characteristics of the eECM. Studies show that stiffness mediates peripheral nerve regeneration through YAP/TAZ regulation of SCs phenotype (Xu et al., 2021). The permeability of scaffold affects the nutrient mass transport, cellular infiltration (such as, macrophage, Schwann cell, fibroblast, endothelial cells), and axonal outgrowth (Varley et al., 2016; Zhang et al., 2019b). Therefore, the stiffness and permeability of the scaffold is a crucial factor for nerve regeneration in early stages.

In the current study, we used micro-CT to acquire high-resolution and high-contrast images of the microstructures of the peripheral nerve eECM (Figure 2). The micro-CT measured area of the endoneurium was larger than that measured from the SEM image, possibly because the micro-CT images lost part of the endoneurium edge as a result of the threshold setting. To the best of the authors’ knowledge, we are the first to obtain detailed images of the human nerve eECM structure. We also obtained the young’s modulus of human peripheral nerve, which is about 38.5 MPa. Compared with other studies, the nerve tissue obtained is relatively soft, which may be due to the relatively long time *in vitro* (Figure 5) (Zhang et al., 2019b; Bartlett et al., 2020). Permeability is widely used to characterize scaffolds for Tissue Engineering (TE) applications as it gives information about the structure porosity, pore size, tortuosity and pore interconnectivity which have an important impact in cell seeding and proliferation (Santos et al., 2020). In this study, we also quantified the porosity, pore size, tortuosity of eECM (Table 2). After reconstruction, the permeability of the three-dimensional microstructure of the nerve eECM analyzed by μ-CFD was $3.10\pm 1.91\times 10^{-12}$ m^2^. The permeability of the fascicles was $2.19\pm 1.09\times 10^{-12}$ m^2^, reflecting the reasonable accuracy of the image-based μ-CFD model.

The limitations of this study mainly include (Robinson, 2022): this study only analyzed the mixed nerves, lacking the analysis of sensory nerves and motor nerves, and the control group (Tiwari et al., 2022); Peripheral nerve has viscoelasticity, and only tensile test cannot accurately reflect its biomechanical characteristics. It is better to use dynamic mechanical analysis for measurement (Wieringa et al., 2018); This study did not verify that bionic permeability is conducive to nerve regeneration *in vivo* and *in vitro* (Li et al., 2021); The data obtained in this experiment are difficult to construct biomimetic scaffolds on the current biological manufacturing technology, and only stay in the theoretical design.

In summary, to the best of the authors’ knowledge, this study is the first of its kind to use micro-CT to obtain high-resolution and high-contrast images of the peripheral nerve eECM, thus enabling the reconstruction of realistic 3D μ-FEA and μ-CFD models for characterizing young’s modulus and permeability through correlations with the experimental results from natural human nerve eECM structures. Analysis of the microstructure and biomechanical characteristics of peripheral nerve eECM would extend the current biomechanical knowledge of nerves. This study is anticipated to provide a fundamental basis for the optimal design of peripheral nerve grafts and/or scaffolds and potentially impacting the regenerative medicine.

## Data Availability

The original contributions presented in the study are included in the article/Supplementary Material, further inquiries can be directed to the corresponding authors.

## Abbreviations

eECM, endoneurium extracellular matrix; micro-CT, microscopic computed tomography; CFD, computational fluid dynamics; SAVR, surface area-to-volume ratio; PNIs, peripheral nerve injuries; ECM, extracellular matrix; NGCs, nerve guidance conduits; IFD, iodine and freeze-dry; ROI, region of interest; RVE, representative volume element; ESD, equivalent spherical diameter; ANA, acellular nerve allograft.
